# In Vitro Synergistic Effects of Selected Essential Oil Components Against Clinical *Acinetobacter baumannii* Strains

**DOI:** 10.3390/molecules31162888

**Published:** 2026-08-19

**Authors:** Magdalena Szemraj, Barbara Kot, Kamila Olszowiec, Zuzanna Walczak, Małgorzata Piechota, Małgorzata Witeska, Monika Sienkiewicz

**Affiliations:** 1 Department of Pharmaceutical Microbiology and Microbiological Diagnostics, Medical University of Lodz, 90-419 Łódź, Poland; magdalena.szemraj@umed.lodz.pl (M.S.); kamila.olszowiec@umed.lodz.pl (K.O.); zuzanna.walczak@student.umed.lodz.pl (Z.W.); monika.sienkiewicz@umed.lodz.pl (M.S.); 2 Institute of Biological Sciences, Faculty of Exact and Natural Sciences, University of Siedlce, 14 Bolesława Prusa Str., 08-110 Siedlce, Poland; malgorzata.piechota@uws.edu.pl; 3 Department of Animal Environment Biology, Institute of Animal Science, Warsaw University of Life Sciences—SGGW, Ciszewskiego 8, 02-786 Warsaw, Poland; malgorzata_witeska@sggw.edu.pl

**Keywords:** *Acinetobacter baumannii*, essential oil components, synergistic interactions, fractional inhibitory concentration (FICI), multidrug-resistant bacteria

## Abstract

Multidrug-resistant *Acinetobacter baumannii* isolates pose a threat to healthcare systems worldwide. The antibacterial activity of essential oil components (EOCs)—linalool, geraniol, eugenol, citronellol, 1,8-cineole, carvacrol, and *trans*-anethole—was evaluated both alone and in combinations against multidrug-resistant *A. baumannii* clinical strains from wound infections, and against two reference strains. The minimum inhibitory concentrations (MICs) and minimum bactericidal concentrations (MBCs) of EOCs were determined using the broth microdilution method. The interactions between EOCs were investigated using the checkerboard microdilution method. Carvacrol and eugenol showed the strongest inhibitory effects against the tested strains. The MICs and MBCs of carvacrol were 1.70–3.39 mg/mL. The MICs of eugenol were 4.10–16.41 mg/mL, while the MBCs were 4.10–32.81 mg/mL. MIC/MBC analysis showed that all tested EOCs had bactericidal activity against *A. baumannii* in vitro; however, some required higher concentrations. Most EOC mixtures showed synergistic interactions. The combination of eugenol and carvacrol induced the strongest synergistic effect, leading to the greatest reduction in the MICs of both compounds (MIC of carvacrol decreased by 8–30-fold, while the MIC of eugenol decreased by 16–64-fold). Our results indicate that some EOCs, especially when combined, exhibit significant antibacterial activity against *A. baumannii*.

## 1. Introduction

*Acinetobacter baumannii* has become a major threat to healthcare systems worldwide over the past few decades, frequently causing hospital-acquired infections [1,2]. Its high adaptability enables survival in natural environments, such as soil and water, as well as in clinical settings, where it readily colonizes abiotic surfaces, medical devices, and human skin, thereby facilitating persistence and transmission within hospital environments [3,4]. *A. baumannii* is responsible for a wide spectrum of infections, including artificial ventilation-associated pneumonia, bloodstream, wound and burn, or urinary tract infections [3,5]. These infections most likely affect immunocompromised patients, particularly those requiring invasive medical procedures or prolonged hospitalization. Reported mortality rates are especially high in cases of pneumonia and bacteremia, often exceeding 30–50% when etiological factors are multidrug-resistant isolates [6,7].

Clinical isolates of *A. baumannii* exhibit increasing resistance to commonly used antibiotics, including carbapenems, which have traditionally been used to treat infections caused by multidrug-resistant strains [6,8]. Owing to this alarming resistance profile, the World Health Organization has designated carbapenem-resistant *A. baumannii* strains as critical priority pathogens requiring the development of new therapeutic strategies [9]. In many cases, the clinical efficacy of available antibiotics is limited or unpredictable, and resistance to last-resort agents such as colistin, tigecycline, and cefiderocol is increasingly reported [6,10]. These trends highlight an urgent need to develop alternatives or complementary antimicrobial methods that enhance the effectiveness of existing treatment options. In this context, essential oils (EOs) and their individual bioactive components (EOCs) have arisen with growing scientific interest.

EOs are complex mixtures of volatile, low-molecular-weight secondary metabolites of plants, predominantly composed of terpenes, terpenoids, and phenylpropanoids [11,12]. In contrast to conventional antibiotics, which typically target a single cellular process, EOCs exhibit multi-target modes of action. This characteristic may reduce the likelihood of rapid resistance development, potentially providing advantages in the treatment of infections caused by multidrug-resistant pathogens [11]. The antimicrobial activity of essential oil components was demonstrated against a broad range of Gram-positive and Gram-negative bacteria, including *Staphylococcus aureus*, *Escherichia coli*, *Klebsiella pneumoniae* and *Pseudomonas aeruginosa* [13,14,15]. Importantly, studies suggest that selected EOCs also exhibit activity against *A. baumannii*, either as individual agents or as adjuvants enhancing the activity of conventional antimicrobials [16,17]. These compounds, particularly terpenoids and phenylpropanoids, display diverse biological activities, including disruption of bacterial membrane integrity, interference with metabolic processes, modulation of oxidative stress, and inhibition of virulence-associated mechanisms [13,15,18].

The compounds investigated in this work belong to the main natural agents with antimicrobial activity. Carvacrol, a phenolic monoterpene, and eugenol, a phenylpropanoid, are among the most potent EOCs [13]. Their activity results in increased cell membrane permeability and dissipation of membrane potential, leading to the leakage of ions and macromolecules, and subsequently, bacterial cell death. These effects are often accompanied by disruption of bacterial energy metabolism and induction of oxidative stress [18,19]. In contrast, monoterpene alcohols, such as geraniol and linalool, as well as oxygenated monoterpenes, such as 1,8-cineole, and aromatic ethers, such as *trans*-anethole, generally exhibit weaker antimicrobial activity when used individually [13,20]. However, these compounds may play a key role in combination with phenolic compounds, facilitating the intracellular accumulation of other substances by increasing cell membrane permeability or reducing efflux efficiency [20,21]. Such interactions provide a mechanistic basis for the synergistic effects often observed in essential oil mixtures [12,15].

The EOCs selected for this study were chosen to represent different chemical classes. These compounds differ in their chemical structures, intrinsic antimicrobial potency, and proposed mechanisms of action. Such diversity provides an appropriate model for investigating whether combining EOCs with complementary properties can enhance antibacterial activity. This could occur through synergistic interactions. As a result, the concentrations required for bacterial inhibition may be reduced, potentially minimizing toxicity-related limitations associated with higher doses.

Synergism may enhance overall antimicrobial efficacy and partially overcome established resistance mechanisms.

The aim of this study was to evaluate the antimicrobial activity of selected EOCs against *A. baumannii* reference strains and clinical isolates, and to assess their synergistic interactions.

## 2. Results

### 2.1. Activity of Individual Essential Oil Components

The antimicrobial activity of individual essential oil components against *A. baumannii* was assessed by determining their MICs, MBCs, and the corresponding MBC/MIC ratios (Table 1).

Selected raw data from MIC and MBC determinations are provided in the Supplementary Materials.

**Figure 1:**
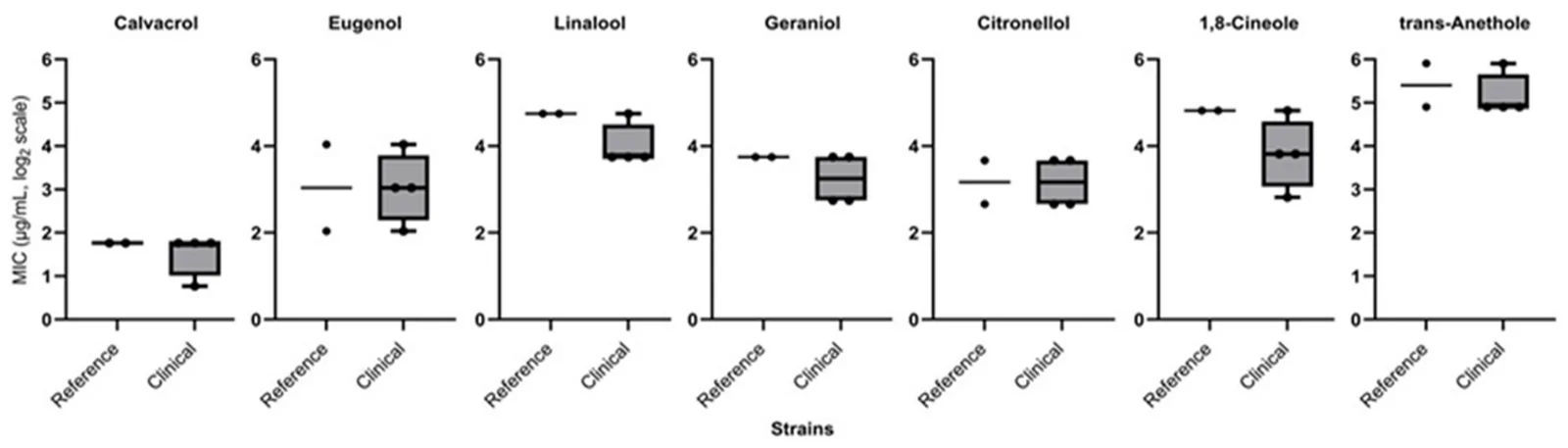
Minimum inhibitory concentration values of essential oil components for reference and clinical *A. baumannii* strains. MIC values (mg/mL) are presented as box-and-whisker plots comparing reference and clinical strains, displayed on a logarithmic scale. Boxes represent medians and interquartile ranges; whiskers indicate minimum and maximum values, and individual points correspond to single strains.

For all components and investigated strains, the calculated MBC/MIC ratios did not exceed 4, indicating bactericidal activity according to established microbiological criteria. In several cases, the MBC equaled the MIC, yielding an MBC/MIC ratio of 1, suggesting rapid bactericidal action following growth inhibition. Although all compounds demonstrated bactericidal activity in vitro, their antimicrobial potential varied. Carvacrol showed the highest bactericidal activity against *A. baumannii* with the lowest MICs (Figure 1).

**Figure 2:**
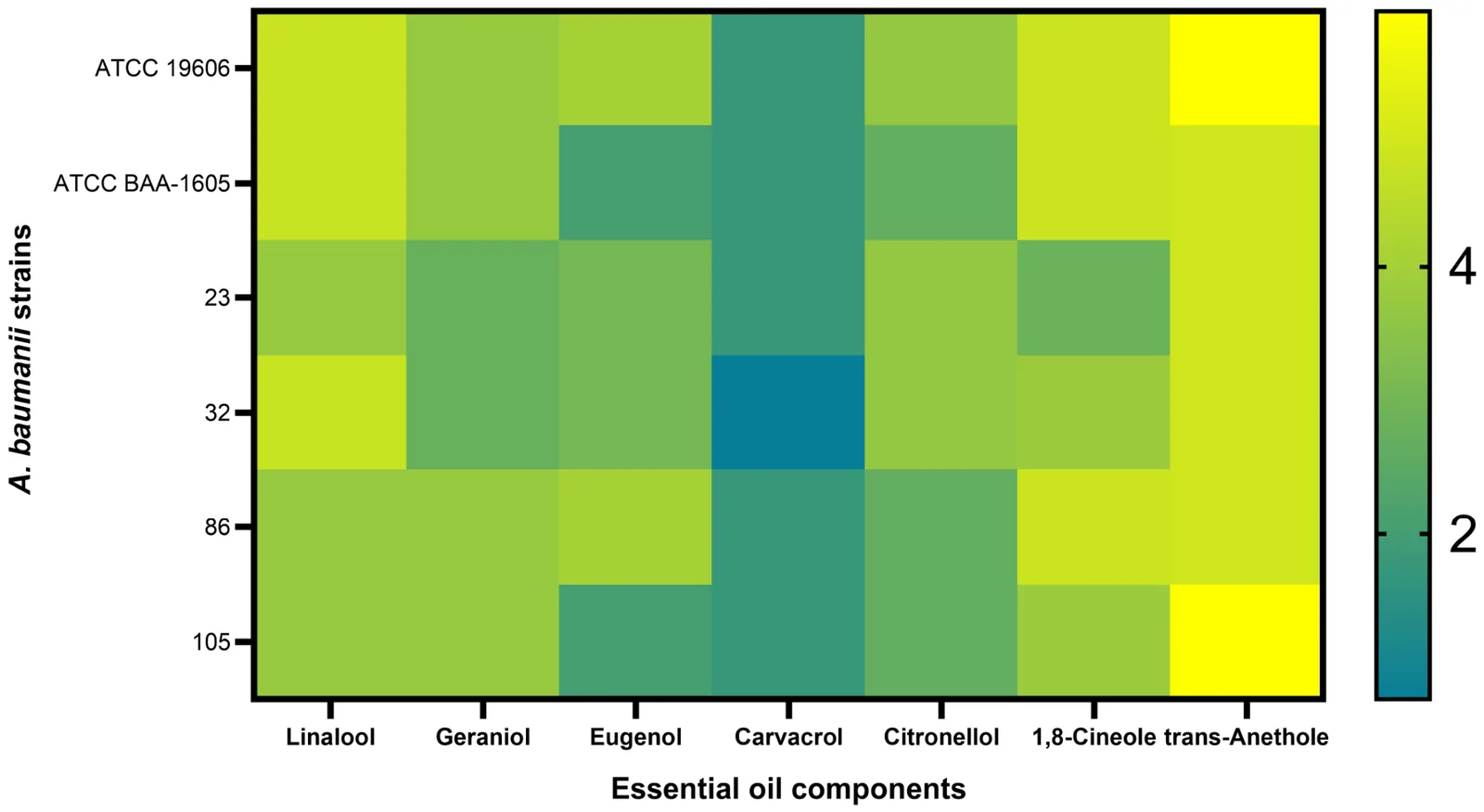
Heatmap representation of the MIC values of investigated EOCs against *Acinetobacter baumannii* strains. MIC values (mg/mL) were log_2_-transformed prior to visualization to facilitate comparison across a wide concentration range. Rows represent individual *A. baumannii* isolates, whereas columns correspond to tested EOCs. Color intensity reflects MIC magnitude: darker colors indicate lower MIC values (greater antimicrobial activity), while lighter colors indicate higher MIC values (lower antimicrobial activity). Each cell represents the MIC value for a specific strain–component pair.

The MIC and MBC of carvacrol against the tested strains ranged from 1.70 mg/mL to 3.39 mg/mL, and the MBC/MIC ratio was 1 (Table 1). The strains’ susceptibility was more varied for eugenol, which inhibited bacterial growth at concentrations ranging from 4.10 mg/mL to 16.41 mg/mL, and bactericidal concentrations ranged from 4.10 mg/mL to 32.81 mg/mL (Table 1). The remaining components tested also showed bactericidal activity against *A. baumannii* but required higher concentrations (Table 1). The MIC values of the tested compounds against *A. baumannii* differed significantly, which is illustrated by Figure 2 showing a heatmap of MICs for all investigated strains. Carvacrol and eugenol demonstrated the highest antibacterial activity against the tested *A. baumannii*, while 1,8-cineole, *trans*-anethole, and linalool required higher concentrations to inhibit the growth of the tested strains. The tested DMSO concentrations had no effect on the growth of the *A. baumannii* strains used in this study.

### 2.2. Comparison of Reference Strains and Clinical Isolates of A. baumannii Susceptibility to Essential Oil Components

A comparison of the MIC values of individual essential oil components for the reference and clinical A. baumannii strains is presented in Figure 1. In the case of linalool, geraniol and 1,8-cineole, the reference strains required higher concentrations to inhibit growth compared to the clinical strains. Despite the varying sensitivity of the assessed reference and clinical strains to EOCs, statistical analysis showed that these differences were not statistically significant (Mann–Whitney U test, *p* > 0.05).

### 2.3. Synergistic Interactions Between Essential Oil Components Against A. baumannii

**Figure 3:**
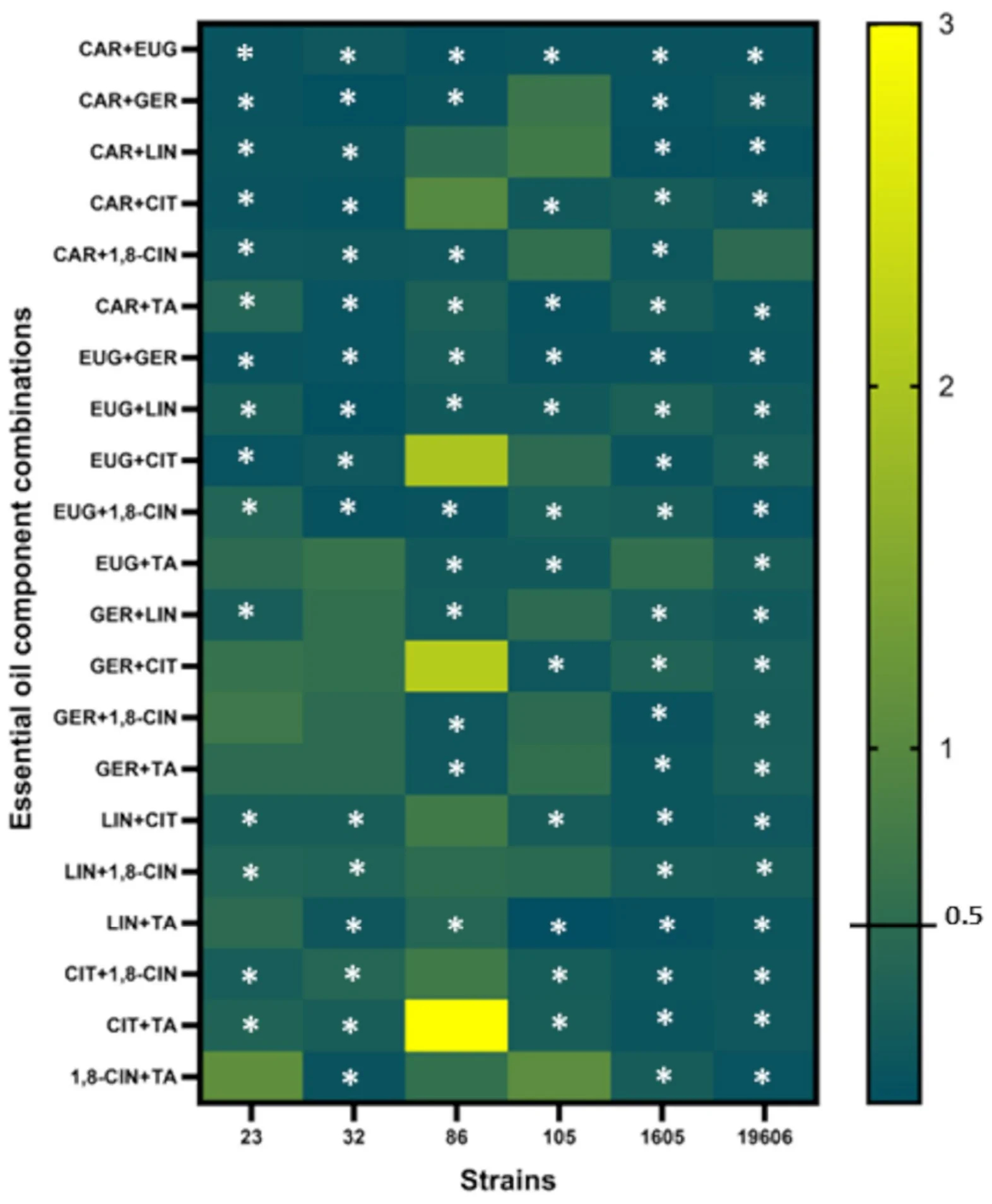
Heatmap of fractional inhibitory concentration index values showing interactions between essential oil components against *Acinetobacter* strains. Color intensity represents the magnitude of FICI values, with darker colors indicating lower FICI values (stronger synergistic interactions) and lighter colors indicating higher FICI values. Interactions were considered synergistic when the FICI was <0.5. The threshold value for synergistic interactions (FICI = 0.5) is indicated on the color scale. Synergistic interactions (FICI < 0.5) are indicated by asterisks (*) in the heatmap. Each cell corresponds to the FICI value for a specific compound combination–strains pair. CAR, carvacrol; EUG, eugenol; GER, geraniol; LIN, linalool; 1,8-CIN, 1,8-cineole; CIT, citronellol; TA, *trans*-anethole.

Synergistic interactions between EOCs were assessed using the fractional inhibitory concentration index (FICI) (Figure 3).

**Figure 4:**
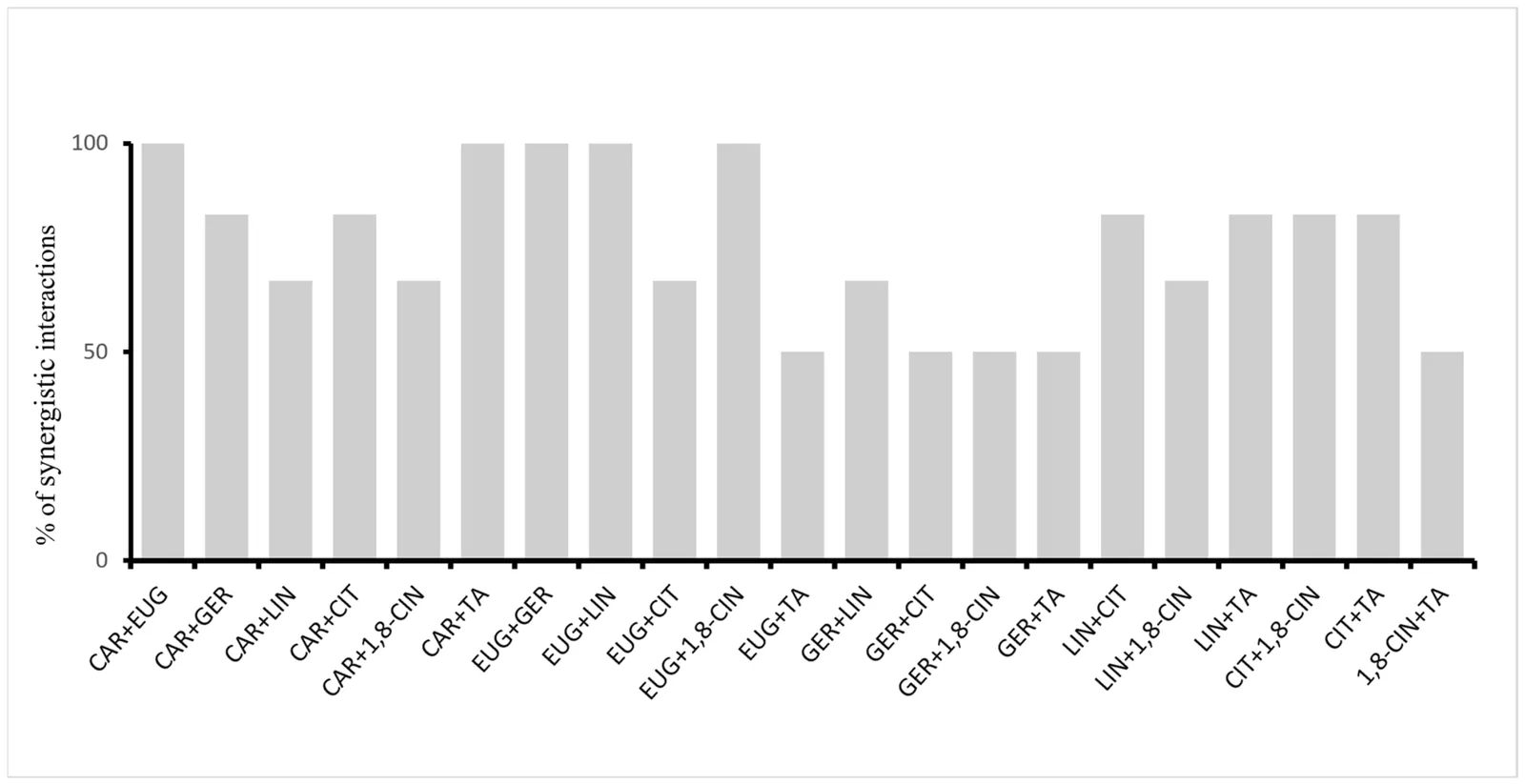
Percentage of strains exhibiting synergistic interactions (FICI < 0.5) for the tested combinations of essential oil components against *A. baumannii* strains. CAR, carvacrol; EUG, eugenol; GER, geraniol; LIN, linalool; 1,8-CIN, 1,8-cineole; CIT, citronellol; TA, *trans*-anethole.

Carvacrol in mixture with eugenol showed a synergistic effect for all strains. Similarly, the combination of eugenol with geraniol, linalool or 1,8-cineole, and also citronellol with 1,8-cineole exhibited synergistic activity against all strains tested (Figure 4).

Mixtures of eugenol with *trans*-anethole, geraniol with citronellol, 1,8-cineole, or *trans*-anethole and 1,8-cineole with *trans*-anethole also showed synergistic activity, but only against 50% of the tested strains. The remaining mixtures of tested components showed synergistic effects against 65–80% of the strains (Figure 4). The percentage of synergistic interactions was calculated as the proportion of strains for which a given EOC combination yielded a FICI value < 0.5 relative to the total number of tested strains (*n* = 6).

**Figure 5:**
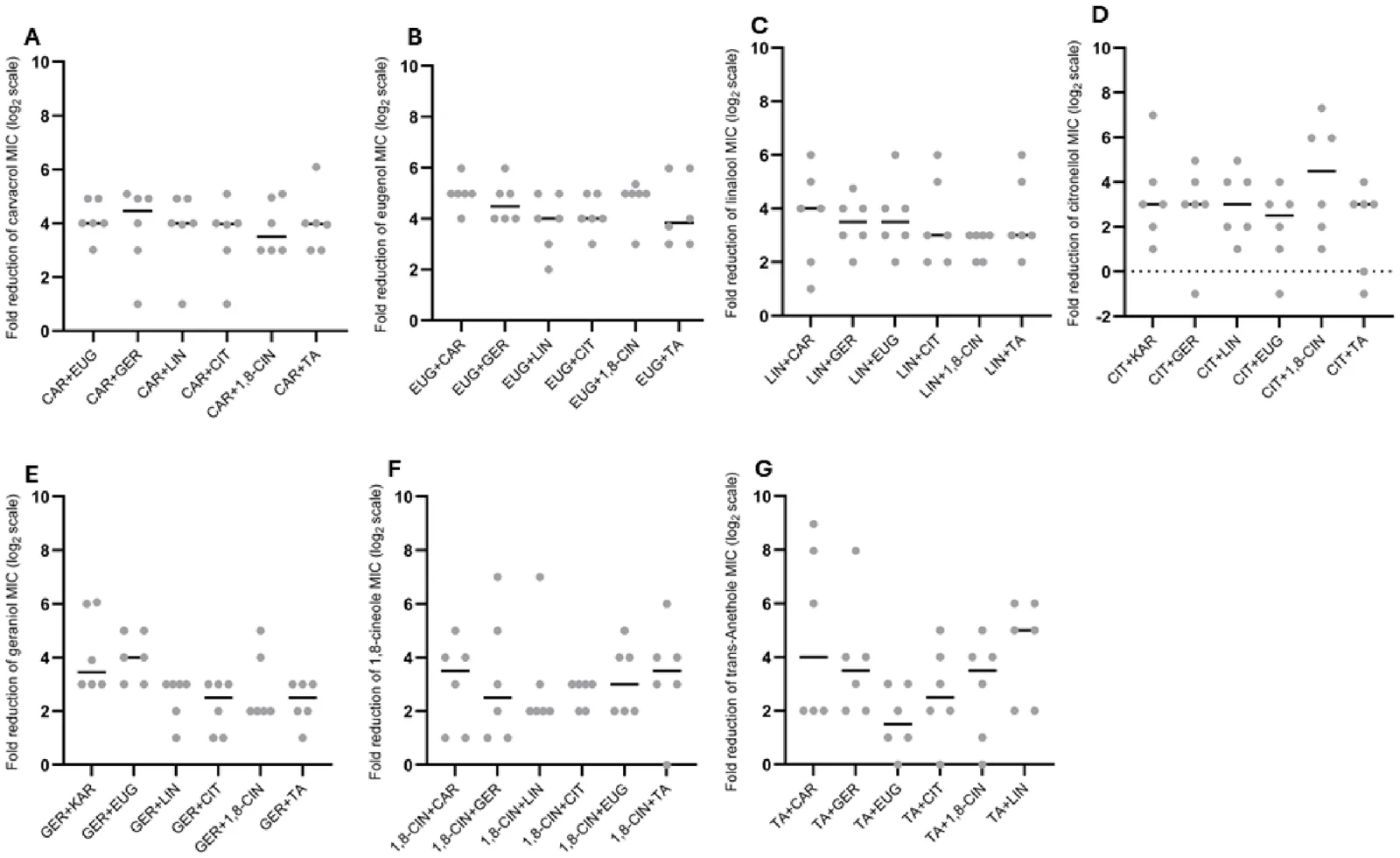
Effective reduction in concentrations of essential oil components in mixtures exhibiting antibacterial activity against *A. baumannii* strains. The reduction in the minimum inhibitory concentration of individual EOCs in binary combinations relative to monotherapy is shown as log_2_-fold reduction values. Panels (**A**–**G**) correspond to carvacrol (**A**), eugenol (**B**), linalool (**C**), citronellol (**D**), geraniol (**E**), 1,8-cineole (**F**), and *trans*-anethole (**G**). Each point represents an individual *Acinetobacter* isolate, and horizontal lines indicate median values. Higher log_2_-fold reduction values reflect a stronger effective concentration reduction.

In addition to FICI-based interaction classification, the degree of reduction in inhibitory component concentrations in synergistic mixtures was assessed, expressed as a log_2_-fold reduction in MIC values relative to monotherapy (Figure 5).

The magnitude of MIC reduction varied depending on the essential oil compounds (EOCs) and their combinations. Of the tested combinations, carvacrol and eugenol-based combinations resulted in significant decreases in MICs for the tested strains. The combination of eugenol and carvacrol showed the strongest synergistic effect, resulting in the greatest reduction in the MICs in all tested strains. In this combination, the MIC of carvacrol decreased by 8–30-fold, corresponding to a 3–5 log_2_-fold reduction, while the MIC of eugenol decreased by 16–64-fold, corresponding to a 4–6 log_2_-fold reduction (Figure 5A,B). Similarly, the combination of eugenol and geraniol resulted in a significant reduction in MIC values: 16–32-fold (4–6 log_2_-fold reduction) for eugenol and 8–32-fold (3–5 log_2_-fold reduction) for geraniol (Figure 5B). In contrast, combinations containing 1,8-cineole or *trans*-anethole resulted in relatively small reductions in concentration (Figure 5F,G). Significant strain-dependent variability was observed across all combinations, indicating that the effective concentration-reduction potential of essential oil combinations was not uniform. For all tested strains, significant MIC reductions were observed with various essential oil combinations. In particular, significant effects were observed for carvacrol in combination with eugenol, 1,8-cineole, or *trans*-anethole; for geraniol in all combinations tested; for linalool in combination with 1,8-cineole; and for combinations of 1,8-cineole with carvacrol, eugenol, geraniol, or citronellol, as well as for *trans*-anethole in combination with carvacrol or geraniol (Wilcoxon one-sample test, *p* < 0.05). Negative log_2_-fold reductions were observed for a small number of strain–combination pairs. These values indicate that the MIC of a given EOC in the combination was higher than its MIC when tested alone. These results likely reflect strain-dependent variability in susceptibility and highlight that not all combinations exert the same degree of concentration-reducing effect on all strains.

Overall, these results demonstrate that while the degree of interaction varies across strains and combinations, selected essential oil combinations are capable of enhancing antimicrobial efficacy by reducing the effective concentrations required for bacterial inhibition (Figure 5A–G).

## 3. Discussion

The spread of multidrug-resistant (MDR) strains of *A. baumannii*, including carbapenem-resistant (CRAB), limits available treatment options. Therefore, there is an urgent need for alternative or complementary treatment strategies. For this purpose, essential oils and their individual components are being investigated [22,23], and our present research aligns with this trend. We evaluated the antibacterial activity of seven selected essential oil components—linalool, carvacrol, eugenol, geraniol, citronellol, 1,8-cineole, and *trans*-anethole, both individually and in combination, against four clinical strains of *A. baumannii* isolated from wound infections, and against two reference strains. All clinical strains were multidrug-resistant, including two carbapenem-resistant isolates. All tested EOCs showed bactericidal activity against both reference and clinical *A. baumannii* strains, with the MBC often equal to the MIC. Carvacrol was the most potent component (MIC 1.70–3.39 mg/mL), while *trans*-anethole showed the weakest monotherapy activity (MIC 30–60 mg/mL). These results are consistent with previous studies showing that carvacrol had the strongest in vitro activity against *A. baumannii*, whereas less polar monoterpenes/ethers were bactericidal at higher concentrations [17,22,24]. Carvacrol’s MIC/MBC values against *A. baumannii* varied across strains. In the study by Tapia-Rodríguez et al. [22], the values were 0.3/0.6 mg/mL, and carvacrol demonstrated strong antibiofilm activity. In contrast, Jahangiri et al. [25] reported higher MIC/MBC values of 3.2/6.4 mg/mL. In the present study, eugenol showed moderate antibacterial activity against the tested *A. baumannii* strains, with MIC values ranging from 4.10 to 16.41 mg/mL. Comparable antibacterial activity of eugenol or eugenol-rich clove oil against *A. baumannii* was reported in previous studies. Mahboubi et al. [26] obtained MIC values of *Eugenia caryophyllata* essential oil ranging from 2 to 16 mg/mL against clinical isolates of *A. baumannii*. Increased antibacterial efficacy was observed when the oil was combined with amikacin, indicating a beneficial effect of combined therapy. In contrast, more recent studies on colistin-resistant *A. baumannii* strains reported lower MIC values of eugenol (approximately 0.4 mg/mL) [27]. These results show that the therapeutic potential of eugenol is strain-dependent and may increase when combined with other antibacterial substances. Geraniol, linalool, citronellol, and 1,8-cineole exhibited higher MIC values in the present study (approximately 6–30 mg/mL), indicating limited antibacterial activity against *A. baumannii* when used individually. This observation is consistent with previous reports indicating that non-phenolic monoterpenoids exhibit weaker direct activity against Gram-negative pathogens due to a reduced ability to disrupt their cell membranes compared to phenolic compounds. However, despite their moderate efficacy when used individually, some of these compounds, particularly geraniol, may play an important role in modifying antibiotic resistance in *A. baumannii*. Kim et al. [28] demonstrated that geraniol significantly increased the antibiotic efficacy in clinical isolates of *A. baumannii* by lowering their MIC values, probably due to inhibition of efflux-dependent resistance mechanisms. Importantly, low concentrations of geraniol did not exhibit cytotoxic or hemolytic effects, confirming its usefulness as an antibiotic adjuvant [28]. Similarly, linalool and 1,8-cineole show limited inhibitory activity against *A. baumannii* [29]. In the case of 1,8-cineole and citronellol, studies show that they increase cell membrane permeability, suggesting their use in synergistic systems but not in monotherapy [30,31,32]. Data on the antibacterial activity of *trans*-anethole against *A. baumannii* are limited. *Trans*-anethole exhibits moderate inhibitory activity when used individually, particularly against Gram-negative bacteria [32]. In this study, *trans*-anethole showed the weakest antibacterial activity among the compounds tested, with growth inhibition observed only at the highest concentrations tested (MIC values reaching approximately 30 mg/mL, depending on the isolate), indicating limited standalone efficacy against *A. baumannii.* The activity of EOCs against reference and clinical strains did not differ significantly. Notably, no clear association was observed between carbapenem resistance and susceptibility to the tested EOCs. Synergistic interactions were observed in both CRAB and non-CRAB isolates, suggesting that the antibacterial activity of EOCs may be largely independent of conventional antibiotic resistance mechanisms. Although the number of clinical isolates examined was limited, these findings support the potential usefulness of EOCs against highly resistant *A. baumannii* strains. Clinical strains often exhibit additional adaptive traits. *Acinetobacter* is a pathogen known for its ability to rapidly acquire and express multidrug resistance mechanisms. Differential activity of the EOCs against *A. baumannii* strains with different resistance profiles was demonstrated by Latib et al. [33]. The MIC ranges (0.78–12.5 mg/mL) indicate that susceptibility to EOCs varies with strain characteristics [33]. This is also confirmed by our studies. Synergy occurred across many EOC combinations used, but no single pair was universally synergistic across all strains. This demonstrates that synergy is not a fixed property of a pair of compounds and depends on both the chemical nature of the compounds and the characteristics of the tested strains [24]. The observed differences in the degree of synergy among the tested essential oil components (EOCs) may be related to their distinct physicochemical properties, functional groups, and cellular mechanisms of action. In general, essential oil components exert antimicrobial effects through multi-target mechanisms, including disruption of bacterial membranes, interference with quorum sensing, induction of oxidative stress, and inhibition of resistance mechanisms such as efflux pumps [13,15,18]. The combination of compounds affecting different cellular targets increases the likelihood of synergistic interactions. Among the pairs we tested, combinations involving carvacrol and eugenol generally demonstrated the greatest improvement in antibacterial efficacy. A key determinant of antimicrobial activity is the presence of specific functional groups, particularly phenolic hydroxyl groups. These observations are consistent with the well-documented membrane-disruptive properties of phenolic compounds such as carvacrol and eugenol [34,35,36]. The strong membrane-disruptive properties reported for these compounds may contribute to the high degree of synergism observed in the present study for combinations involving carvacrol and eugenol.

Carvacrol–eugenol combinations produced some of the strongest and most repeatable synergistic results. Although eugenol alone demonstrated only moderate activity against *A. baumannii*, its combination with carvacrol resulted in a significant decrease in MIC values in many strains. Similarly, carvacrol in combination with 1,8-cineole resulted in a lower MIC for carvacrol, despite 1,8-cineole’s limited efficacy alone. Non-phenolic monoterpenes such as limonene, α-pinene, and eucalyptol lack polar functional groups and therefore exhibit weaker intrinsic antimicrobial activity. Their primary role may be indirect, involving modulation of membrane permeability to facilitate the uptake of more active antimicrobial compounds. For example, limonene has been shown to disrupt the outer membrane and lipopolysaccharide structure, thereby enhancing the uptake of other antimicrobial agents. This explains why compounds with low monotherapy activity (e.g., 1,8-cineole) can still exhibit synergistic interactions when combined with phenolic EOCs [30,32]. This interpretation is consistent with our findings, in which 1,8-cineole exhibited relatively high MIC values but contributed to synergistic interactions, albeit with a lower magnitude of MIC reduction compared to combinations of highly active phenolic compounds.

This is particularly significant because oxygenated monoterpenes such as 1,8-cineole are often rejected due to high MICs. The results demonstrate that weak activity against a given strain does not preclude synergy when combined with a high-activity substance, such as carvacrol. *trans*-anethole demonstrated the highest MIC and MBC values among all compounds tested, confirming its limited antibacterial activity when used alone. The weak antibacterial activity of trans-anethole observed in this study may also be attributed to its chemical structure, as it lacks a free phenolic hydroxyl group. Although it belongs to phenylpropanoids, its higher hydrophobicity and limited hydrogen-bonding capacity reduce its ability to directly disrupt bacterial membranes [32].

Despite this limited intrinsic activity, trans-anethole demonstrated synergistic interactions, particularly with carvacrol. One possible explanation is that trans-anethole may alter membrane fluidity and lipid organization, thereby facilitating the penetration of more active compounds, as suggested in previous studies on aromatic phenylpropanoids. Consequently, trans-anethole may act as a membrane-modulating compound, indirectly contributing to the overall antibacterial effect.

The importance of such complementary mechanisms is supported by composition–activity relationship analyses, which indicate that compounds such as carvacrol, eugenol, and certain terpenoids are the main contributors to antimicrobial activity, whereas others, including monoterpene hydrocarbons and oxygenated monoterpenes, may enhance activity indirectly through synergistic interactions [12,15]. These findings suggest that synergism is not solely determined by the potency of individual compounds but by their functional interplay.

Similarly, the combination of eugenol and geraniol resulted in a significant reduction in MIC values. Such interactions may also be associated with previously reported effects of certain terpenoids, including geraniol, on bacterial resistance mechanisms, particularly those involving efflux-mediated resistance [28].

Additionally, other monoterpene alcohols tested in this study, including linalool and citronellol, may act through similar mechanisms. These compounds can disrupt membrane integrity and alter membrane fluidity, which increases bacterial permeability and facilitates the activity of more potent EOCs. Although their intrinsic antibacterial activity is limited, they play an important role in synergistic systems. Differences in the degree of synergism observed between linalool and citronellol may be related to their molecular structure, polarity, and interactions with membrane lipids. Linalool has also been shown to influence biofilm formation and quorum sensing, further supporting its modulatory role [29].

In our study, we analyzed not only whether synergy occurred but also by how much the MIC was reduced with different EOC combinations. This is important because the toxicity of essential oils is often concentration-dependent [25,28]. The nature of synergistic interactions revealed by FICI analysis reflects the complex, multi-target mechanisms by which essential oil components exert their antimicrobial effects. Phenolic compounds primarily disrupt membrane integrity, whereas other compounds may enhance permeability or interfere with intracellular processes. Thus, the degree of synergism depends not only on the presence of interaction but also on the relative contribution of each compound to the overall antimicrobial effect. Importantly, these differences are reflected in the extent of MIC reduction observed in this study (Figure 5), where combinations containing phenolic compounds led to markedly greater MIC reductions than those containing non-phenolic components.

The degree of synergism observed in this study also appears to depend on the physicochemical compatibility of the compounds. Synergistic effects are more pronounced when compounds differ in polarity and mechanism of action, allowing simultaneous disruption of multiple cellular processes. In contrast, combinations of structurally and mechanistically similar compounds may result in weaker or strain-dependent effects. This may explain the lower and more variable synergistic effects observed for combinations involving non-phenolic compounds such as 1,8-cineole or trans-anethole.

This effect provides a mechanistic explanation for the higher frequency of synergistic interactions observed with combinations involving these compounds. On the other hand, oxygenated monoterpenes generally exhibit weaker antibacterial activity and limited membrane-disrupting capacity, which may explain their lower efficacy [32]. Reduction in concentration is particularly important from a translational perspective because it can reduce cytotoxicity, volatility limitations, and selective pressure for resistance development [37,38]. Reduction in effective concentration complements FICI analysis by providing additional information regarding the practical relevance of synergistic interactions and their potential translational value [39].

An additional strength of the present study is the systematic evaluation of all pairwise combinations among seven selected essential oil components against both reference and clinically relevant MDR *A. baumannii* strains. Such comparative analyses remain limited in the literature, particularly for combinations involving *trans*-anethole and 1,8-cineole.

Several limitations of this study should be acknowledged. First, antibacterial activity and synergistic interactions were evaluated solely under in vitro conditions. Second, the study did not investigate the molecular mechanisms underlying the observed synergistic effects, such as membrane permeabilization, efflux pump inhibition, or oxidative stress induction. The scientific literature provides general information on the mechanism of action of the most active components of essential oils, but studies designed for specific research systems with the tested bacterial strains are needed. For example, it is known that carvacrol’s potent activity is due to its multiple mechanisms of action, including interaction with cell membrane components, leading to membrane disruption, leakage of contents, and thus cell lysis. Other mechanisms include inhibition of efflux pumps, inhibition of bacterial motility, and inhibition of membrane ATPases [40]. Eugenol’s antimicrobial activity was also found to include cell membrane damage and inhibition of cellular enzymes; it is also responsible for inducing oxidative stress, and it can also bind to bacterial DNA [41]. Linalool, in turn, has an inhibitory effect on ATPases and dehydrogenases, disrupting the respiratory chain and inhibiting the synthesis of cellular energy, and may also induce the formation of reactive oxygen species (ROS), which causes peroxidation of cell membrane lipids [42]. Finally, cytotoxicity toward mammalian cells and antibiofilm efficacy were not assessed. Therefore, further studies are required to determine the therapeutic applicability and safety of the most promising EOC combinations.

Finally, the strain-dependent variability observed in the present study likely reflects differences in membrane composition, permeability, and resistance mechanisms among clinical isolates. The effectiveness of synergistic combinations depends on the ability of EOCs to overcome specific barriers present in a given strain, such as altered lipid composition or increased efflux activity. Therefore, antimicrobial synergy should be considered a context-dependent phenomenon influenced by both compound structure and bacterial phenotype.

## 4. Materials and Methods

### 4.1. Bacterial Strains

A total of four clinical *A. baumannii* strains obtained from wounds and two reference strains of *A. baumannii* ATCC 19606 and *A. baumannii* ATCC BAA-1605 were included in this study. The tested strains came from the Department of Pharmaceutical Microbiology and Microbiological Diagnostics, Medical University of Lodz collection. The clinical strains were obtained in 2024 as part of routine diagnostic microbiology conducted in hospitals and were identified by the MALDI-TOF method. Antimicrobial resistance and the presence of carbapenem resistance genes were previously determined [8]. All strains were multidrug-resistant, including two with phenotypic carbapenem resistance (Table 2).

### 4.2. Chemicals

Seven EOCs were used in this study: carvacrol (≥98% purity; Pol-Aura, Zawroty, Poland), eugenol (>99% purity; TCI, Tokyo, Japan), geraniol (>95% purity; Pol-Aura, Zawroty, Poland), 1,8-cineole (≥98.5 purity; Pol-Aura, Zawroty, Poland), citronellol (>95% purity; Thermor Fisher Scientific, Waltham, MA, USA), linalool (≥97% purity; Pol-Aura, Zawroty, Poland) and *trans*-anethole (98% purity; Pol-Aura, Zawroty, Poland). Each EOC was diluted in dimethyl sulfoxide (DMSO) (Sigma-Aldrich, Darmstadt, Germany) at a concentration not exceeding 2% (*v*/*v*) for testing antibacterial activity.

### 4.3. Evaluation of the Antimicrobial Activity

The minimum inhibitory concentrations (MICs) and minimum bactericidal concentrations (MBCs) of EOCs against the tested bacteria were evaluated using the broth microdilution method in sterile 96-well microtiter plates. The broth microdilution testing was performed in accordance with the recommendations of the European Committee on Antimicrobial Susceptibility Testing (EUCAST) [43]. Stock solutions of EOCs were prepared in DMSO and subsequently diluted in Mueller–Hinton broth (MHB; Sigma-Aldrich, Darmstadt, Germany) to obtain final testing concentrations ranging from 250 to 0.9 mg/mL. Each well was inoculated with 100 µL of the prepared EOC dilution and 100 µL of the bacterial suspension in MHB, resulting in a final inoculum density of about 5 × 10^5^ colony-forming units per milliliter (CFU/mL). The study also included the growth control of each strain in the presence of serial two-fold dilutions of DMSO in MHB without the addition of the tested EOCs. Following incubation for 18 h at 37 °C under aerobic conditions, MIC values were determined as the lowest concentration of the tested compound that inhibited visible bacterial growth. Growth was assessed spectrophotometrically at 595 nm using a BioTek EPOCH microplate spectrophotometer (BioTek Instruments, Winooski, VT, USA). To obtain MBC values, 10 µL aliquots were collected from wells without visible growth and plated onto Brain Heart Infusion agar (BHI; bioMérieux, Marcy-l’Étoile, France). The plates were incubated for 24 h at 37 °C. The MBCs were determined as the lowest concentrations of the tested compounds that led to the absence of bacterial growth, corresponding to ≥99.9% reduction in the initial inoculum. The bactericidal or bacteriostatic nature of EOCs was evaluated by calculating the MBC/MIC ratios. Ratios ≤ 4 were considered bactericidal, whereas ratios > 4 were considered bacteriostatic, in accordance with established microbiological criteria widely used in the studies [44]. Each assay was performed three times under the same conditions.

### 4.4. The Checkerboard Assay

To determine interactions between selected EOCs, the checkerboard microdilution method was used, as previously described [45]. Each well of a sterile 96-well microtiter plate contained a final volume of 200 µL, consisting of 50 µL of each essential oil component made in two-fold serial dilutions in Mueller–Hinton broth (MHB). Subsequently, 100 µL of MHB containing the bacterial suspension of 1 × 10^6^ CFU/mL was added to each well. Plates were incubated for 18 h at 37 °C. Growth of the tested bacteria was assessed spectrophotometrically by measuring optical density at 595 nm, and MIC values were determined for individual components and their combinations. For each combination, the fractional inhibitory concentration (FIC) was set as the ratio of the MIC of an EOC in combination to the MIC of the same EOC tested alone. The fractional inhibitory concentration index (FICI) was obtained as the sum of the individual FIC values of both components, according to the following formulas:

$$FIC=\frac{{MIC}_{EOC}\;in\;combination}{{MIC}_{EOC\;}alone}$$

FICI = FICEOC1 + FICEOC2

Interactions were interpreted as synergistic (FICI < 0.5), additive (0.5 ≤ FICI ≤ 1.0), indifferent (1.1 < FICI ≤ 4.0), or antagonistic (FICI > 4.0).

Each assay was performed three times under the same conditions.

### 4.5. Statistical Analysis

Comparisons of MIC values between reference and clinical strains were conducted using the nonparametric Mann–Whitney U test, due to the small sample size and nonnormal distribution of the data. A two-tailed test was applied, and a *p*-value < 0.05 was considered statistically significant. The reduction in EOC concentrations in combinations was assessed by calculating the fold reduction in MIC values of individual essential oil components in combination relative to monotherapy. A one-sample Wilcoxon signed-rank test was used to compare fold-reduction values against the no-effect reference value of 1 (corresponding to no reduction in MIC), with each strain serving as its own control. For the statistical analysis, STATISTICA 13.1 PL software was applied (StatSoft 2016, Cracow, Poland). Due to the limited number of strains, additional inter-group statistical comparisons were not performed.

## 5. Implications, Limitations, and Future Directions

Our results support the notion that EOCs, particularly phenolic compounds, are most effective in rationally designed combinations rather than monotherapies. Furthermore, they highlight the importance of broad strain panels in the study of antimicrobial synergy. However, the study is limited to in vitro analysis and does not address the potential cytotoxicity, stability, or pharmacokinetic limitations associated with EOCs. Future studies should aim to elucidate the molecular mechanisms underlying the observed synergistic interactions.

## 6. Conclusions

In summary, this study highlights the importance of evaluating essential oil components within rationally designed combination strategies against *A. baumannii*. Although several of the tested compounds demonstrated activity at high concentrations, their concentration-reduction effects in synergistic combinations highlight the significant potential to enhance antimicrobial efficacy while mitigating key translational constraints such as cytotoxicity, volatility, and selective pressures that promote resistance development. Our research fits into the strategy to develop mixtures with synergistic effects against multidrug-resistant bacteria. The significant strain-dependent variability observed in clinical isolates further emphasizes that antimicrobial synergy cannot be predicted using single reference strains alone. Instead, evaluation in diverse clinical settings is necessary to capture biologically relevant patterns of interaction. Concentration-reduction results position essential oil component combinations as promising ways to aid in the search for alternative or adjunctive treatment strategies for multidrug-resistant *A. baumannii*. These studies contribute to filling the therapeutic gap caused by the decreased effectiveness of conventional antibiotic therapies (topical administration of mixtures or as an addition to implantable medical devices) and also indicate the possibility of using these compounds as natural agents eliminating *A. baumannii* from the hospital environment, including various objects and surfaces in the hospital environment, thus limiting the spread of drug resistance of *A. baumannii*. Therefore, further mechanistic and in vivo studies are necessary to translate these in vitro observations into clinically applicable approaches.

## Figures and Tables

**Table 1:** The antimicrobial activity of individual essential oil components against *A. baumannii* strains.

EOCs	Isolates ID	MIC (mg/mL)	MBC (mg/mL)	MBC/MIC Ratio
Carvacrol				
	23	3.39	3.39	1
	32	1.70	1.70	1
	86	3.39	3.39	1
	105	3.39	3.39	1
	ATCC BAA 1605	3.39	3.39	1
	ATCC 19606	3.39	3.39	1
Eugenol				
	23	8.20	8.20	1
	32	8.20	8.20	1
	86	16.41	16.41	1
	105	4.10	4.10	1
	ATCC BAA 1605	4.10	8.20	2
	ATCC 19606	16.41	32.81	2
Linalool				
	23	13.44	13.44	1
	32	26.88	26.88	1
	86	13.44	26.88	2
	105	13.44	26.88	2
	ATCC BAA 1605	26.88	57.75	2
	ATCC 19606	26.88	26.88	1
Geraniol				
	23	6.72	13.44	2
	32	6.72	13.44	2
	86	13.44	53.75	4
	105	13.44	13.44	1
	ATCC BAA 1605	13.44	13.44	1
	ATCC 19606	13.44	26.88	2
Citronellol				
	23	12.69	12.69	1
	32	12.69	12.69	1
	86	6.34	12.69	2
	105	6.34	12.69	2
	ATCC BAA 1605	6.34	6.34	1
	ATCC 19606	12.69	12.69	1
1,8-Cineole				
	23	7.06	14.11	2
	32	14.11	28.22	2
	86	28.22	28.22	1
	105	14.11	28.22	2
	ATCC BAA 1605	28.22	28.22	1
	ATCC 19606	28.22	56.44	2
*trans*-anethole				
	23	29.94	59.88	2
	32	29.94	59.88	2
	86	29.94	59.88	2
	105	59.88	119.75	2
	ATCC BAA 1605	29.94	59.88	2
	ATCC 19606	59.88	59.88	1

EOCs—essential oil components; MIC—minimum inhibitory concentration; MBC—minimum bactericidal concentration.

**Table 2:** The clinical *A. baumannii* strains used in this study.

Strain ID	Resistance Phenotypes	Carbapenem Resistance Genes
23	TZP, CAZ, FEP, GM, AK, TOB, CIP, SXT	*bla*_OXA-23_
32	CAZ, GM, AK, CIP, LEV, SXT	*bla*_OXA-23_
86	TZP, CAZ, IPM, MEM, GM, AK, TOB, CIP, LEV, SXT	*bla*_OXA-23_, *bla*_OXA-40_
105	TZP, CAZ, FEP, IPM, MEM, GM, AK, TOB, CIP, LEV, SXT	*bla*_OXA-23_, *bla*_OXA-40_

TZP—piperacillin with tazobactam; CAZ—ceftazidime; FEP—cefepime; IPM—imipenem; MEM—meropenem; GM—gentamicin; AK—amikacin; TOB—tobramycin; CIP—ciprofloxacin; LEV—levofloxacin; SXT—trimethoprim plus sulfamethoxazole.

## Data Availability

The original contributions presented in this study are included in the article and Supplementary Materials. Further inquiries can be directed to the first author.

## Supplementary Materials

The following supporting information can be downloaded at https://www.mdpi.com/article/10.3390/molecules31162888/s1.
