# Supplementary material for: In Vitro Synergistic Effects of Selected Essential Oil Components Against Clinical Acinetobacter baumannii Strains
Source: Molecules. 2026 Aug 19;31(16):2888. doi: 10.3390/molecules31162888 (PMC13515966; doi:10.3390/molecules31162888)
Supplement: Supplementary file 1 [file molecules-31-02888-s001.zip › molecules-4422494-supplementary.pdf]

Supplementary Materials

In Vitro Synergistic Effects of Selected Essential Oil Components Against Clinical *Acinetobacter baumannii* Strains

Magdalena Szemraj [1], Barbara Kot [2,*], Kamila Olszowiec [1], Zuzanna Walczak [1], Małgorzata Piechota [2], Małgorzata Witeska [3] and Monika Sienkiewicz [1]

1 Department of Pharmaceutical Microbiology and Microbiological Diagnostics, Medical University of Lodz, 90-419 Łódź, Poland; magdalena.szemraj@umed.lodz.pl (M.S.); kamila.olszowiec@umed.lodz.pl (K.O.); zuzanna.walczak@student.umed.lodz.pl (Z.W.); monika.sienkiewicz@umed.lodz.pl (M.S.)

2 Institute of Biological Sciences, Faculty of Exact and Natural Sciences, University of Siedlce, 14 Bolesława Prusa Str., 08-110 Siedlce, Poland; malgorzata.piechota@uws.edu.pl

3 Department of Animal Environment Biology, Institute of Animal Science, Warsaw University of Life Sciences—SGGW, Ciszewskiego 8, 02-786 Warsaw, Poland; malgorzata_witeska@sggw.edu.pl

* Correspondence: barbara.kot@uws.edu.pl

The Supplementary Materials provide raw data supporting the determination of minimum inhibitory concentrations (MICs) and minimum bactericidal concentrations (MBCs) for selected essential oil components against clinical *Acinetobacter baumannii* strains 23 and 32. Figure S1–S2 contain raw OD595 measurements obtained in broth microdilution assays for carvacrol and citronellol. Figures S3–S4 present MBC determinations based on subculturing aliquots from wells showing no visible growth.

Software Version	3.11.2019
User Name:	System Administrator
Experiment File Path:	
Protocol File Path:	
Plate Number	Plate 4
Date	9.04.2025
Time	09:17:07
Reader Type:	Epoch
Reader Serial Number:	21110320
Reading Type	Reader
**Procedure Details**	
Plate Type	96 WELL PLATE (Use plate lid)
Eject plate on completion	
Read	Absorbance Endpoint
	Full Plate
	Wavelengths: 595
	Read Speed: Normal, Delay: 100 msec, Measurements/Data Point: 8
**Results**	
Actual Temperature:	0

	1	2	3	4	5	6	7	8	9	10	11	12	
A	0,131	0,107	0,124	0,144	0,148	0,152	0,148	0,151	0,159	0,152	0,158	0,121	595
B	0,216	0,155	0,213	0,186	0,2	0,186	0,188	0,644	1,01	1,086	1,13	1,212	595
C	0,141	0,169	0,201	0,192	0,182	0,184	0,178	0,669	1,088	1,114	1,06	1,208	595
D	0,141	0,216	0,185	0,211	0,184	0,181	0,19	0,543	0,985	1,029	1,034	1,153	595
E	0,151	0,161	0,207	0,194	0,183	0,188	0,188	0,184	1,112	1,143	1,135	1,151	595
F	0,13	0,185	0,223	0,302	0,195	0,198	0,191	0,164	1,068	1,096	1,077	1,092	595
G	0,104	0,143	0,209	0,21	0,194	0,186	0,183	0,132	1,075	1,067	1,106	1,157	595
H	0,105	0,118	0,123	0,109	0,111	0,125	0,11	0,125	0,122	0,12	0,105	0,107	595

Figure S1. Raw OD_{595} measurements from broth microdilution assays performed with carvacrol against clinical *A. baumannii* strains 23 and 32.

Row A represents the carvacrol dilution control. Rows B–D correspond to strain 23, whereas rows E–G correspond to strain 32. Column 1 represents the medium control, and column 12 corresponds to the bacterial growth control. Columns 2–11 contain two-fold serial dilutions of carvacrol (108.69, 54.31, 27.16, 13.58, 6.79, 3.39, 1.70, 0.85, 0.425 and 0.212 mg/mL). The MIC values of carvacrol were 3.39 mg/mL for strain 23 and 1.70 mg/mL for strain 32.

Software Version	3.11.2019
User Name:	System Administrator
Experiment File Path:	
Protocol File Path:	
Plate Number	Plate 2
Date	18.09.2025
Time	09:39:45
Reader Type:	Epoch
Reader Serial Number:	21110320
Reading Type	Reader
**Procedure Details**	
Plate Type	96 WELL PLATE (Use plate lid)
Eject plate on completion	
Read	Absorbance Endpoint
	Full Plate
	Wavelengths: 595
	Read Speed: Normal, Delay: 100 msec, Measurements/Data Point: 8
**Results**	
Actual Temperature:	0

	1	2	3	4	5	6	7	8	9	10	11	12	
A	0,116	0,182	0,122	0,101	0,14	0,145	0,127	0,149	0,15	0,152	0,142	0,128	595
B	0,1	0,212	0,2	0,172	0,186	0,738	1,045	1,147	1,1	1,054	1,034	1,099	595
C	0,098	0,183	0,177	0,167	0,178	0,715	0,924	1,073	1,107	1,046	1,032	1,08	595
D	0,156	0,189	0,18	0,173	0,189	0,741	0,854	1,052	1,069	1,095	1,091	1,074	595
E	0,247	0,215	0,179	0,184	0,186	0,542	0,691	0,844	0,972	0,994	0,999	0,95	595
F	0,251	0,23	0,197	0,181	0,189	0,421	0,701	0,908	1,063	1,158	1,173	1,216	595
G	0,1	0,211	0,184	0,188	0,217	0,608	0,728	0,763	0,799	0,818	0,802	0,937	595
H	0,098	0,138	0,164	0,164	0,153	0,152	0,164	0,159	0,168	0,166	0,158	0,113	595

Figure S2. Raw OD_{595} measurements from broth microdilution assays performed with citronellol against clinical *A. baumannii* strains 23 and 32.

Row A represents the citronellol dilution control. Rows B–D correspond to strain 23, whereas rows E–G correspond to strain 32. Column 1 represents the medium control, and column 12 corresponds to the bacterial growth control. Columns 2–11 contain two-fold serial dilutions of citronellol (101.5, 50.75, 25.38, 12.69, 6.34, 3.17, 1.59, 0.79, 0.40 and 0.20 mg/mL). The MIC of citronellol was 12.69 mg/mL for both strains.

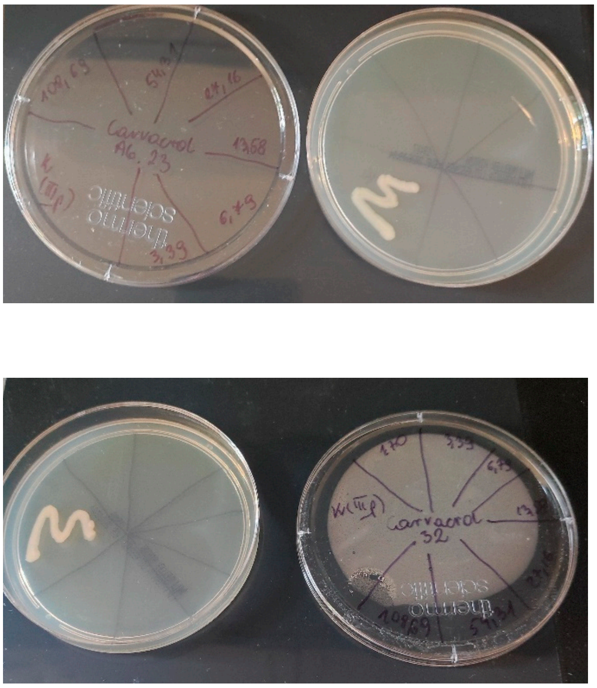

Figure S3. MBC determination for clinical *A. baumannii* strains 23 and 32 following exposure to carvacrol. Aliquots from wells corresponding to the MIC concentration and higher concentrations were subcultured onto Brain Heart Infusion agar. Growth was observed in the corresponding positive growth controls for both strains. No bacterial growth was observed at the MIC concentration, indicating that the MIC and MBC values were identical for both strains (3.39 mg/mL for strain 23 and 1.70 mg/mL for strain 32).

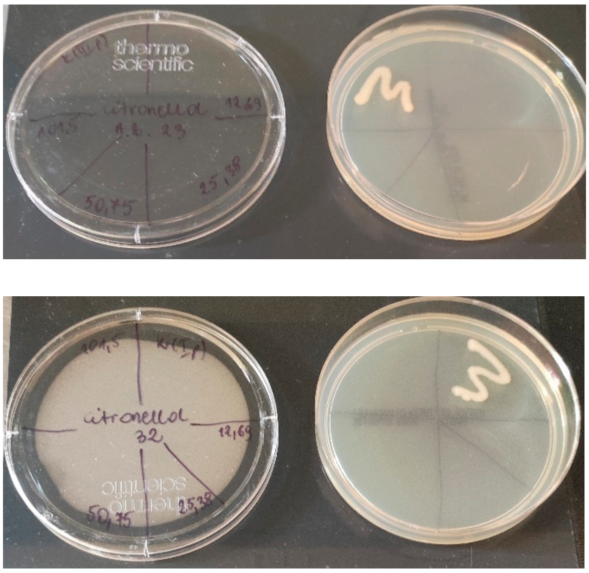

Figure S4. MBC determination for clinical *A. baumannii* strains 23 and 32 following exposure to citronellol.

Aliquots from wells corresponding to the MIC concentration (6.34 mg/mL) and higher concentrations were subcultured onto Brain Heart Infusion agar. Growth was observed in the corresponding positive

growth controls for both strains. No bacterial growth was observed at the MIC concentration for either strain, indicating that MIC and MBC values were identical (12.69 ng/mL).
